# A Generalized Pyramid Matching Kernel for Human Action Recognition in Realistic Videos

**DOI:** 10.3390/s131114398

**Published:** 2013-10-24

**Authors:** Jun Zhu, Quan Zhou, Weijia Zou, Rui Zhang, Wenjun Zhang

**Affiliations:** 1 Institute of Image Communication and Network Engineering, Shanghai Jiao Tong University, Shanghai 200240, China; E-Mails: zhujun.sjtu@gmail.com (J.Z.); zouweijia@sjtu.edu.cn (W.Z.); zhang_rui@sjtu.edu.cn (R.Z.); 2 College of Telecommunications and Information Engineering, Nanjing University of Posts and Telecommunications, Nanjing 210003, China; E-Mail: quan.zhou@njupt.edu.cn

**Keywords:** video analysis, human action recognition, pyramid matching kernel, kernel-based classification method

## Abstract

Human action recognition is an increasingly important research topic in the fields of video sensing, analysis and understanding. Caused by unconstrained sensing conditions, there exist large intra-class variations and inter-class ambiguities in realistic videos, which hinder the improvement of recognition performance for recent vision-based action recognition systems. In this paper, we propose a generalized pyramid matching kernel (GPMK) for recognizing human actions in realistic videos, based on a multi-channel “bag of words” representation constructed from local spatial-temporal features of video clips. As an extension to the spatial-temporal pyramid matching (STPM) kernel, the GPMK leverages heterogeneous visual cues in multiple feature descriptor types and spatial-temporal grid granularity levels, to build a valid similarity metric between two video clips for kernel-based classification. Instead of the predefined and fixed weights used in STPM, we present a simple, yet effective, method to compute adaptive channel weights of GPMK based on the kernel target alignment from training data. It incorporates prior knowledge and the data-driven information of different channels in a principled way. The experimental results on three challenging video datasets (*i.e.*, Hollywood2, Youtube and HMDB51) validate the superiority of our GPMK *w.r.t.* the traditional STPM kernel for realistic human action recognition and outperform the state-of-the-art results in the literature.

## 1. Introduction

Recognition of human actions, e.g., running, fighting and shooting balls, is an increasingly important research topic in the fields of video sensing, analysis and understanding [1–3]. It applies to a wide range of computer vision applications, such as video surveillance [4,5], human-computer interaction [6,7], sports video analysis [8,9] and content-based video retrieval [10,11]. Although promising progress has been achieved for human action recognition in constrained scenarios [12,13], recognition accuracy remains unsatisfactory for realistic videos (e.g., TV, movies and Internet videos) [14–16]. This is mainly because they are taken under unconstrained sensing conditions and, thus, suffer from a great number of visual challenges (e.g., object pose, background clutter, camera motion, viewpoint and illumination variations), which result in large intra-class variations and inter-class ambiguities that hinder the improvement of recognition performance in recent vision-based action recognition systems.

To overcome these challenges, massive research efforts have been dedicated to vision-based systems on realistic human action recognition over last few years [9,14–20]. Related work in human action recognition literature can be generally divided into two categories: (1) The first category relies on the technologies of detecting and analyzing human body movement (e.g., kinematic tracking [21], human body pose estimation [22], space-time shape templates [13], *etc.*) in video sequences and, then, performs action recognition on that basis; (2) As an extension of a classic framework in the image classification field [23–27], the second category aims at directly building a holistic feature representation of the video clip for human action recognition, based on *local spatial-temporal features*[28] and the “*bag of words*” (BoW) model [23]. Specifically, we can first extract localized feature points from video clips by using spatial-temporal interest point (STIP) detectors [17,29] or dense sampling methods [20,28] and, then, capture appearance, shape and motion information in the neighborhoods of detected points by feature descriptors [17,20,30–32]. After that, the BoW model calculates the visual word frequency statistics on quantized local feature points, to form a histogram-wise video representation for classification. Compared with the first category of action recognition approaches, the latter one becomes increasingly dominant for realistic human action recognition because of its many advantages: the detected STIPs have a certain degree of invariance to illumination, scale and viewpoint changes; the BoW representation is robust to relatively large intra-class variations of human action patterns in realistic videos; it is easy to be implemented and does not require complicated motion capture or human body analysis technologies.

However, traditional BoW representation fails to capture useful distribution information in the spatial-temporal domain for detected orderless STIPs, which is crucial for the recognition of complex action classes in realistic videos. The success of *spatial pyramid matching* (SPM) [24] in the static image recognition domain [25–27] motivates researchers to incorporate the spatial-temporal layout information of local features for dynamic videos [10,17,19,20,33]. Choi *et al.* [10] extended the SPM technology into the scenario of 3D video clips and proposed a *spatial-temporal pyramid matching* (STPM) kernel for sports video matching and retrieval. Similar to SPM, the STPM is built to find the approximate correspondence of local feature points for a valid similarity metric between two video clips, by accumulating the matched feature points over a sequence of increasingly denser 3D grids in the spatial-temporal domain. Besides, Ni *et al.* [33] proposed a depth-layered spatial-temporal feature pooling method to utilize the depth information of STIPs, which shows boosted recognition performance compared to the conventional STIP BoW method for recognizing human daily activities in RGB-D videos. Moreover, recent research progress [20,34] demonstrates this to be an effective means of improving recognition performance, to combine multiple types of feature descriptors for utilizing their complementarity, and shows state-of-the-art performance on realistic human action recognition.

As shown in [10,24], the SPM/STPM kernel adopts *ad hoc* weights for combining the similarities in different grid granularity levels, which are inversely proportional to the cell width for penalizing the matching of local feature points found in larger cells. In spite of the success of SPM/STPM in many vision tasks, the fixed and predefined weights are not adaptive to the characteristics of training data. In this paper, we propose a new matching kernel to measure the similarity of two video clips, called the *generalized pyramid matching kernel* (GPMK), to leverage heterogeneous visual cues in multiple feature descriptor types and spatial-temporal grid granularity levels. Compared to the STPM kernel, our GPMK mainly has the following two advantages: (1) It extends standard STPM kernel into a more general form (*i.e.*, the STPM kernel presented in [10] can be deemed a special case of our GPMK.) and provides a more flexible fusion of multi-channel information for discrimination, by means of assigning independent weights on different channels of feature descriptor types and spatial-temporal grid granularity levels; (2) We present a simple, yet effective, approach to compute adaptive channel weights of GPMK based on the *kernel-target alignment* [35] from training data, which incorporates the prior knowledge and data-driven information of multiple channels in a principled way. In addition, we show that the proposed GPMK is a valid Mercer kernel, which is a desirable property for facilitating its usage in training kernel-based classifiers [36,37]. Thus, we apply it to the task of human action recognition in realistic videos via a kernel-based support vector machine (SVM) [36] classification framework. The experimental results on three challenging realistic video datasets (i.e., Hollywood2 [14], Youtube [15] and HMDB51 [16]) validate the superiority of our GPMK *w.r.t.* traditional STPM kernel for human action recognition, and show higher classification accuracy than previous action recognition approaches in the literature. In summary, the main contributions of our method are listed below:

- We extend the STPM kernel and present a GPMK as a valid similarity metric between two video clips, which has a more general and flexible form to fuse heterogeneous information in multiple feature types and spatial-temporal grid levels.
- Instead of the predefined and fixed weights used in STPM, we propose a new method to compute the adaptive channel weights of GPMK based on the kernel-target alignment from training data.
- On the basis of the GPMK, we design a human action recognition system for realistic videos via a kernel-based SVM classification framework. The experimental results on three public datasets validate the superiority of our GPMK *w.r.t.* the STPM kernel for realistic human action recognition and outperform the state-of-the-art results in the literature.

The rest of this paper is organized as follows: In Section 2, we elaborate on the kernel-based SVM classification framework for human action recognition in videos. Section 3 introduces the construction of multi-channel BoW representation from local spatial-temporal features. After that, we formally present the GPMK, as well as the method for calculating adaptive channel weights in Section 4. In Section 5, we show experimental results and detailed analysis on the proposed GPMK. Finally, we conclude this paper in Section 6.

## 2. The Kernel-Based SVM Classification Framework

In this paper, we adopt the kernel-based SVM classification framework [36] for human action recognition in videos. Assuming there is a set of *N* video clips (denoted by {x_1_, x_2_, ⋯ , x*_i_*, ⋯ , x*_N_*}) used for training the classifier, let *y_i_* ∈ { − 1,1} denote the binary action class label of video clip x*_i_*. The values of 1 and −1 represent the positive and negative classes respectively, indicating whether the target human action appears in this video clip or not. We use *y* = [*y*_1_, *y*_2_, ⋯ ,*y_i_*, ⋯ , *y_N_*]^T^ to represent the label vector of all training samples accordingly. Then, we build a *N* × *N* kernel matrix **K** from training samples, in which the element of the *i*-th row and the *j*-th column is given by **K***_ij_* = 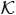
(x*_i_*, x*_j_*). In this paper, 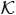
(·, ·) denotes the proposed GPMK, which is a valid kernel function for measuring the similarity between two video clips.

**Figure 1.**
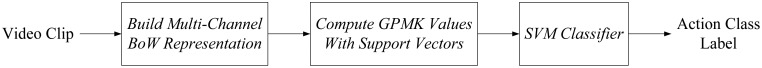
The flowchart of our kernel-based human action recognition system.

In the training stage, we learn a kernel SVM classifier by solving the following convex quadratic programming problem [36]:

(1) $$\begin{array}{cc} \min_{\alpha } & \frac{1}{2}\alpha^{\text{T}}\tilde{\text{K}}\alpha -e^{\text{T}}\alpha \\ \text{s}.\text{t}. & y^{\text{T}}\alpha =0,\;\text{and}\;0\leq \alpha_{i}\leq C,\;i=1,2,\cdots ,N \end{array}$$

where *α* = [*α*_1_, *α*_2_, ⋯ , *α_i_*, ⋯, *α_n_*]^T^ denotes a coefficient vector whose entries are zero except for the support vector samples [36]. Meanwhile, e and *C* represent an *N*-dimensional unit vector and a predefined regularization parameter on training the SVM model, respectively. **K̃** is a *N* × *N* label-augmented kernel matrix, where **K̃***_ij_* = *y_i_y_j_***K***_ij_* for each pair of samples, *i* and *j*. In the testing stage, we compute the classifier's score of a new video clip, x, as follows:

(2) $$f(\text{x})=\sum_{i\in sv}y_{i}\alpha_{i}K(\text{x},{\text{x}}_{i})+b$$

where *sv* and *b* denote the support vector set of training samples and a constant bias term, respectively. Thus, the class label of x can be identified via a sign function *y* = sgn(*f*(x)), where *y* = 1 for *f*(x) ≥ 0 and *y* = − 1, otherwise. In Figure 1, we show the flowchart of kernel-based SVM classification for human action recognition in videos. For a test video clip, we first build a multi-channel BoW representation from the local spatial-temporal features extracted on it and, then, compute its GPMK values *w.r.t.* the learned support vectors of training samples. After that, we can classify this video clip by evaluating its score according to Equation (2). In the following part of this paper, we will elaborate on the multi-channel BoW representation and the GPMK in Sections 3 and 4, respectively.

## 3. Multi-Channel BoW Representation for a Video Clip

Assuming there are *Q* feature descriptor types used in total, let $P_{\text{x}}^{q}(q\;\in \;\{1,2,\cdots ,Q\})$ denote a set of local spatial-temporal feature points extracted from video clip x for the *q*-th feature type. After that, given a dictionary with *M* visual words for each feature type, we discretize the descriptors of local feature points with corresponding visual word indices based on the vector quantization approach, as in [10,23,24]. Concretely, for every point, we compute the Euclidean distance between its feature descriptor vector and each of the visual words in the dictionary and map it to the visual word of minimum distance.

Similar to the STPM [10], let *L* denote the maximum grid granularity level of the spatial-temporal pyramid used. We partition a whole video clip into a sequence of evenly-divided 3D spatial-temporal grids with (*L* + 1) different granularity levels. For each grid granularity level *l* ∈ {0, 1, ⋯, *L*}*,* x is uniformly partitioned by 2*^l^* bins in each of the spatial-temporal dimensions, and thus, a total of *D_l_* = 2*^3l^* subvolumes can be obtained. For each feature type *q* ∈ {1, 2, ⋯ ,*Q*}, we compute the occurrence frequency of visual words in the dictionary for different spatial-temporal subvolumes and obtain several BoW histograms [23] as the video representation used in GPMK. Let ${\text{h}}_{n}^{l}(P_{\text{x}}^{q})={\left[h_{n,1}^{l}(P_{\text{x}}^{q}),h_{n,2}^{l}(P_{\text{x}}^{q}),\cdots ,h_{n,m}^{l}(P_{\text{x}}^{q}),\cdots ,h_{n,M}^{l}(P_{\text{x}}^{q})\right]}^{\text{T}}$ denote an *M*-dimensional vector of the BoW histogram on quantized local feature points in the *n*-th subvolume of grid granularity level *l*, where $h_{n,m}^{l}(P_{\text{x}}^{q})$ is equal to the number of feature points belonging to the *m*-th visual word.

**Figure 2.**
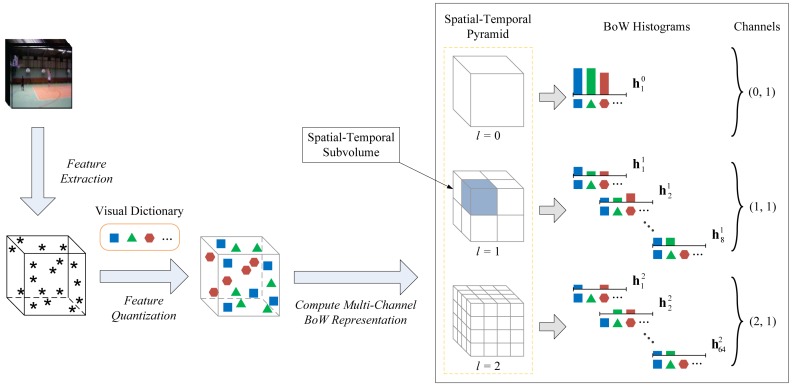
Illustration of building the multi-channel bag of words (BoW) representation for a video clip. In this figure, the local feature points are represented by black stars, and the quantized features are shown with geometrical shape symbols in different colors (e.g., blue square, green triangle and red hexagon), each type of which indicates one visual word. A three-level spatial-temporal pyramid (*i.e.*, *L* = 2) is adopted for demonstration. For clarity, we show the case of using only one feature descriptor type (*i.e.*, *Q* = 1) as an example. For the case of multiple feature descriptor types used, the BoW histograms for the channels of each individual feature type can be computed in a similar way. (Note: the image is best viewed in color with magnification).

In this paper, we name the combination of grid granularity level *l* and feature type index *q* by a term *channel* (*l*, *q*) (We index the channel with a binary group notation for simplicity and intuition). Generally, it includes two aspects as follows: on the one hand, *q* indicates the type of feature descriptor used for capturing certain visual cues of local feature points extracted from the video clip; on the other hand, *l* corresponds to the grid granularity of encoding spatial-temporal distribution information for the quantized features. Thus, a video clip can be represented by a total of *Q* × (*L* + 1) BoW histograms in different feature descriptor types and spatial-temporal grid granularity levels, and we build a multi-channel BoW representation from local spatial-temporal features, which is used in the calculation of GPMK. Figure 2 illustrates the process of building the multi-channel BoW representation for a video clip.

## 4. A Generalized Pyramid Matching Kernel with Adaptive Channel Weights

In this section, we first present the GPMK as a valid similarity metric between two video clips and, then, elaborate on the method of computing adaptive channel weights based on the kernel-target alignment [35].

### 4.1. The Generalized Pyramid Matching Kernel

In this paper, the GPMK is defined as a valid video similarity metric, for leveraging heterogeneous visual cues in multiple feature descriptor types and spatial-temporal grid granularity levels. Similar to STPM, we build the GPMK to find approximate correspondences of local feature points between two video clips, by accumulating the matched feature points over a sequence of increasingly denser 3D grids in the spatial-temporal domain.

Let x and z denote two video clips. At first, we compute the matching degree of these two video clips for each channel, respectively, which is defined by the total number of matched feature points over different visual words and spatial-temporal subvolumes in that channel. In practice, this can be calculated via a histogram intersection kernel (HIK) [38] function in Equation (3).

(3) $$I_{q}^{l}(\text{x},\text{z})=\overset{D_{l}}{\underset{n=1}{\text{∑}}}\overset{M}{\underset{m=1}{\text{∑}}}\min[h_{n,m}^{l}(P_{\text{x}}^{q}),h_{n,m}^{l}(P_{\text{z}}^{q})]$$

The matching degree for channel (*l*, *q*), which is denoted by $I_{q}^{l}$ in Equation (3), between x and z is equal to the sum of the minimum value on each bin of their BoW histograms ${\text{h}}_{n}^{l}(P_{\text{x}}^{q})$ and ${\text{h}}_{n}^{l}(P_{\text{z}}^{q})$ over all of the *D_l_* subvolumes in level *l*.

After that, we construct the GPMK by combining the matching degree values over different channels, to build a unified similarity metric of video clips. Formally, it is defined by Equation (4), which is a weighted sum of $I_{q}^{l}$ over all the channels.

(4) $$\begin{array}{cc} & K(\text{x},\text{z})=\sum_{l=0}^{L}\sum_{q=1}^{Q}\omega_{\left(l,q\right)}{ℐ}_{q}^{l}(\text{x},\text{z})\\ \text{s}.\text{t}. & \forall \;l\;\text{and}\;q,\;\omega_{\left(l,q\right)}\geq 0 \end{array}$$

where *ω*_(*l*,*q*)_ denotes the weight of channel (*l*, *q*) in GPMK.

As an extended form of the SPM/STPM kernel, the proposed GPMK is able to assign independent weight value for each individual channel (*i.e.*, the combination of feature descriptor type and grid granularity level) and provides a more flexible fusion of heterogeneous information for discrimination. As proven in [39,40], the HIK function of calculating ${ℐ}_{q}^{l}$ is positive definite. Besides, we constrain the weight ω_(*l*,*q*)_ to be non-negative for each channel. Thus, the resultant GPMK defined in Equation (4) is a conic sum (*i.e.*, linear combination with non-negative weights) [41] over a series of positive definite kernel functions on individual channels, such that it is a valid Mercer kernel, which facilitates the usage in training SVM classifiers [36,37]. Actually, according to [10], the STPM kernel is a special case of our GPMK that uses *ad hoc* weights by $\omega_{\left(l,q\right)}=\frac{1}{Q\cdot 2^{L}}$ for *l* = 0 and $\omega_{\left(l,q\right)}=\frac{1}{Q\cdot 2^{L-l+1}}$ for other levels *l* ∈ 1, 2, ⋯ , *L*.

### 4.2. Adaptive Channel Weights Based on Kernel-Target Alignment

In this paper, rather than using fixed and predefined weights as in SPM/STPM, we compute adaptive channel weights for GPMK based on the *kernel-target alignment*[35]. Let ${\text{I}}_{q}^{l}$ denote a *N* × *N* kernel matrix of channel (*l*, *q*) on training samples, in which the element of the *i*-th row and the *j*-th column is equal to ${ℐ}_{q}^{l}({\text{x}}_{i},{\text{x}}_{j})$. Besides, we use the ground-truth class label vector, **y**, to compute a target indicator matrix **Y** = yy^T^, which presents an ideal discriminative characteristic on the classification of training samples. For each channel (*l, q*), we calculate the kernel-target alignment value, *s*_(*l*,*q*)_, between ${\text{I}}_{q}^{l}$ and **Y** on training data, which corresponds to the cosine of the angle between those two matrices [35]:

(5) $$s_{\left(l,q\right)}=\frac{{\langle {\text{I}}_{q}^{l},\text{Y}\rangle }_{F}}{\sqrt{{\langle {\text{I}}_{q}^{l},{\text{I}}_{q}^{l}\rangle }_{F}\cdot {\langle \text{Y},\text{Y}\rangle }_{F}}}=\frac{{\langle {\text{I}}_{q}^{l},\text{Y}\rangle }_{F}}{N\sqrt{{\langle {\text{I}}_{q}^{l},{\text{I}}_{q}^{l}\rangle }_{F}}}$$

In Equation (5), 〈**A**, **B**〉*_F_* = *tr*(**A***^T^***B**) represents the Frobenius inner product of two matrices, **A** and **B**.

Actually, *s*_(*l*,*q*)_ works as a simple and intuitive assessment on the discriminative power of channel (*l*, *q*) according to the training data. For instance, a larger alignment value for one channel implies that its kernel matrix is more similar to the target matrix, indicating higher discriminability for classification, and thus, a larger weight should be assigned to that channel. Based on the alignment values obtained from training data for all individual channels, we compute an adaptive weight for each channel (*l*, *q*) in GPMK, via the following soft max activation function:

(6) $$\begin{array}{c} \omega_{\left(l,q\right)}=\frac{\pi_{\left(l,q\right)}e^{\beta s_{\left(l,q\right)}}}{\sum_{l'=0}^{L}\sum_{q'=1}^{Q}\pi_{\left(l^{\prime },q^{\prime }\right)}e^{\beta s_{\left(l^{\prime },q^{\prime }\right)}}}\\ \forall l=0,1,\cdots ,L\;\text{and}\;q=1,2,\cdots ,Q \end{array}$$

where *π*_(*l*,*q*)_ is a non-negative prior term for channel (*l, q*) and *β* is a predefined parameter on the smoothness of soft max activation. In this paper, we set the value of *π*_(*l*,*q*)_ to be consistent with the STPM kernel (*i.e.*, $\pi_{\left(l,q\right)}=\frac{1}{Q\cdot 2^{L}}$ for *l* = 0 and $\pi_{\left(l,q\right)}=\frac{1}{Q\cdot 2^{L-l+1}}$ for other levels *l* = 1, 2, ⋯ , *L*). Thus, the proposed GPMK using weight values defined in Equation (6) would reduce to the standard STPM kernel when *β* = 0.

As observed in Equation (6), the channel weight of GPMK is determined based on two terms: on the one hand, the prior term π_(*l*,*q*)_ represents the preference on the significance of channel (*l, q*); on the other hand, $e^{\beta s_{\left(l,q\right)}}$ is a data-driven term corresponding to the alignment value between the kernel matrix of channel (*l*, *q*) and the target matrix. Instead of the fixed and predefined weights used in SPM/STPM, we compute the channel weights of GPMK in a data-driven manner, which are adaptive to the characteristics of the training data. The parameter, *β,* controls the trade off between the prior term and the data-driven one. Moreover, a larger value of *β* highlights the channels with relatively higher alignment values via the soft max activation function. When the value of *β* increases, the GPMK will assign larger weights to more discriminative channels and shrink weight values of the channels with smaller alignment values through the L1-normalization of *ω*. Thus, our GPMK is able to adaptively select the channels with relatively higher discriminative power according to training data. Besides, observing that the weights defined in Equation (6) are always non-negative and subject to the constraints of Equation (4), the derived GPMK is indeed a valid Mercer kernel, as stated in Section 4.1.

## 5. Experiments

In this section, we evaluate the proposed GPMK on three public action datasets (*i.e.*, Hollywood2 [14], Youtube [15] and HMDB51 [16]) and show its superiority *w.r.t.* the STPM kernel [10] for human action recognition in realistic videos. Besides, we compare it with previous methods in the action classification literature and provide detailed analysis for the GPMK.

### 5.1. The Datasets

In this subsection, we briefly introduce the Hollywood2, Youtube and HMDB51 datasets used in our experiments. They provide realistic video clips for the action recognition benchmark under challenging conditions, such as camera motion, object appearance and pose, object scale, cluttered background, viewpoint and illumination variations, *etc.*

#### 5.1.1. The Hollywood2 Dataset

This dataset is composed of video clips collected from 69 movies, which intends to provide a comprehensive benchmark for human action recognition in realistic scenarios. It consists of 12 human action classes as follows: answering the phone, driving car, eating, fighting, getting out of car, hand shaking, hugging, kissing, running, sitting down, sitting up and standing up. Following common settings in the literature [14,18,20,42], we use the manually verified clean data in our experiments. There are a total of 1,707 video clips divided into a training subset (823 video clips) and a testing subset (884 video clips), which come from different movies. As in [14], we calculate the average precision (AP) of binary classification for each action class separately, and the overall performance is measured by the mean AP (mAP) over all classes.

#### 5.1.2. The Youtube Dataset

**Figure 3.**
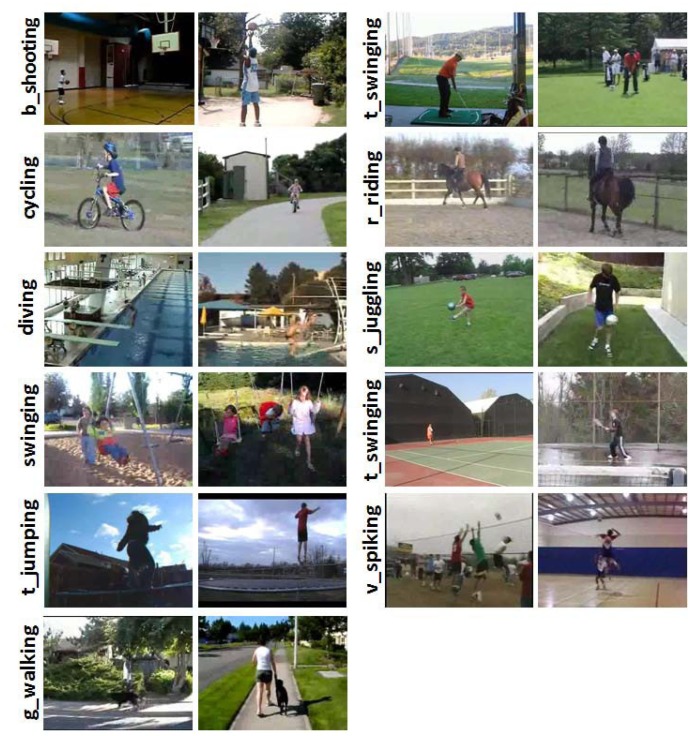
Illustration of the sample frames from video clips for all the 11 human action classes in the Youtube dataset [15]. (Note: the image is best viewed in color).

This dataset is collected from the Internet, which includes a total of 1,168 realistic video clips. It contains 11 human action classes: basketball shooting, biking/cycling, diving, golf swinging, horseback riding, soccer juggling, swinging, tennis swinging, trampoline jumping, volleyball spiking and walking with a dog. Following the original protocol of [15], we adopt the predefined 25-fold leave-one-out cross validation for training and testing, and the performance is measured by the average of per-class classification accuracy over different folds. Figure 3 illustrates the sample frames from video clips for different human action classes in the Youtube dataset.

#### 5.1.3. The HMDB51 Dataset

**Figure 4.**
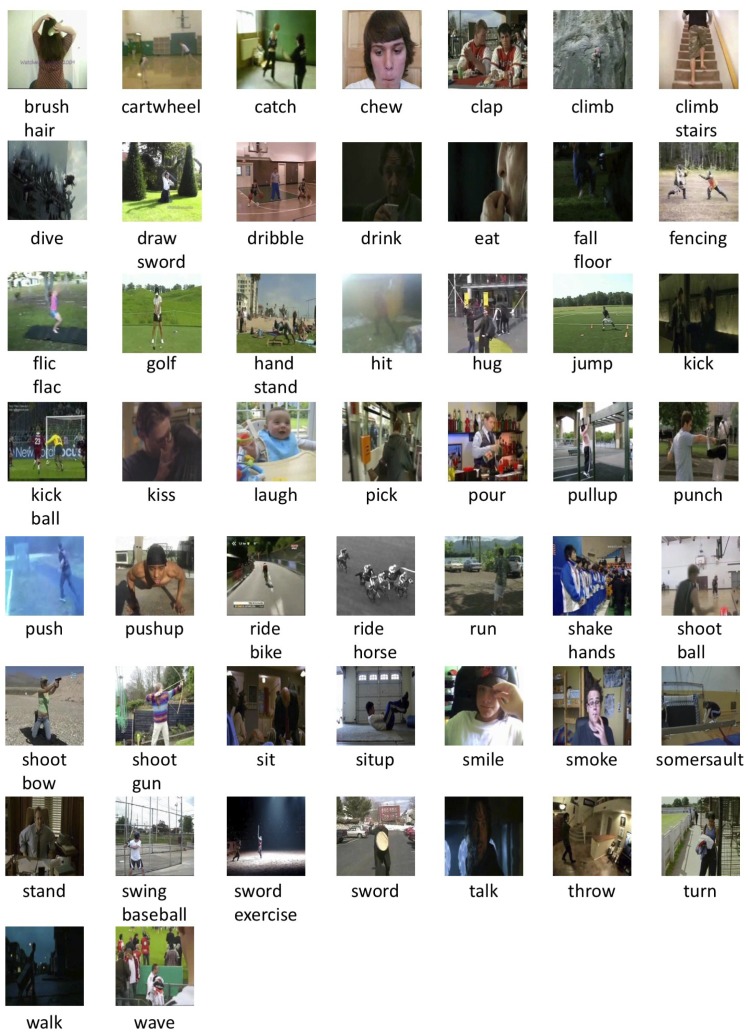
Illustration of the sample frames from video clips for all 51 human action classes in the HMDB51 dataset [16]. (Note: the image is best viewed in color).

This dataset consists of 6,766 video clips distributed into 51 human action classes, which are collected from a variety of realistic video sources, such as movies and Internet videos. It contains the largest number of classes and video clips in the action classification literature. In experiments, we adopt the same three training/testing sample splits released by the authors of [16]. For each split, there are 70 training video clips and 30 testing ones per action class. Besides, we use the original non-stabilized version of video clips in our experiments. Following [16], the recognition performance is measured by the mean value of per-class classification accuracy over all three training/testing splits. Figure 4 illustrates the sample frames from video clips for different human action classes in the HMDB51 dataset.

### 5.2. Experimental Settings

In the feature extraction step, we adopt a dense trajectory detector [20] with four different feature descriptor types (*i.e.*, motion boundary histogram [31], histogram of oriented gradients [17], histogram of optical flow [17] and trajectory shape descriptor [20]) to capture motion, appearance and shape cues of local spatial-temporal feature points. Furthermore, we use mean RGB values as an additional feature type to take advantage of the color information of densely extracted local feature points, and thus, there are a total of five kinds of feature descriptors (*i.e.*, *Q* = 5) used in our experiments. For the feature quantization step, we randomly select 100,000 samples of local feature points from training video clips and then, employ the K-means clustering algorithm [43] on their feature descriptors to build the visual dictionary. Specifically, the obtained cluster centers are used as the visual words in the dictionary. Following the common setting in the action classification literature [17,20,44], we set the number of visual words by *M* = 4, 000 for each feature descriptor type.

In building the GPMK, the value of parameter *β* in Equation (6) is set by 30 for Hollywood2, 20 for Youtube and 10 for HMDB51, respectively. In the classification step, we implement the SVM classifier by the LIBSVM code package [45] and adopt the “one-*vs.*-rest” criterion on multi-class discrimination for Youtube and HMDB51. Specifically, we train a binary SVM classifier for each action class individually, and then, the class label of the testing video is predicted as the one with the highest score output by the corresponding classifier. The regularization parameter, *C*, for training SVM classifiers is constantly set to 8 throughout our experiments.

### 5.3. Performance Evaluation

In this subsection, we evaluate recognition performance on the proposed GPMK and compare it with the STPM kernel. In practice, STPM is used as a baseline method in this paper, which actually corresponds to the special case of *β* = 0 for GPMK. Besides, we compare the result of our GPMK with previous methods in action classification literature.

**Figure 5.**
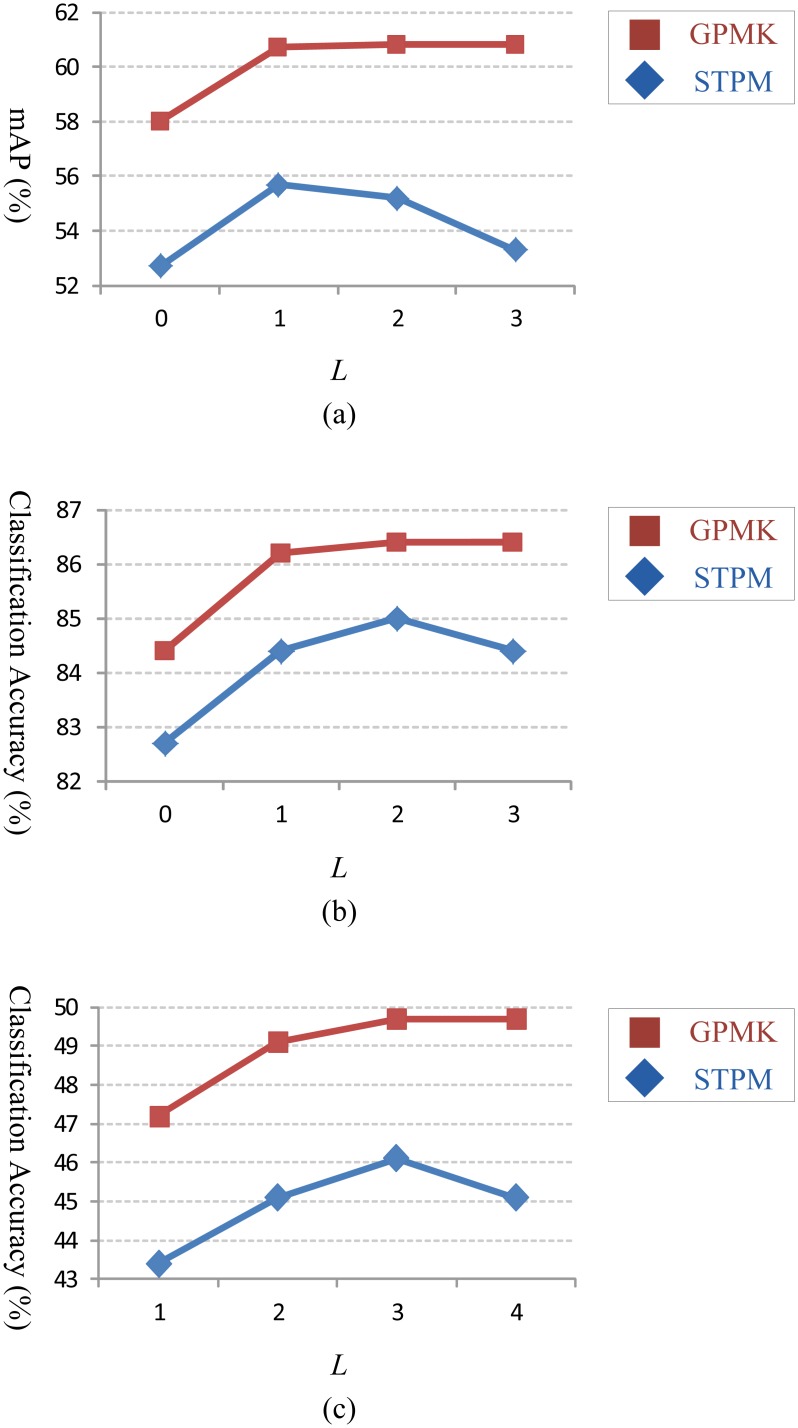
The recognition performance (shown on the Y-axis) *w.r.t.* different values of *L* (shown on the X-axis): (**a**) Hollywood2; (**b**) Youtube; (**c**) HMDB51. In each panel, the red square markers correspond to our generalized pyramid matching kernel (GPMK), and the blue diamond ones represent the spatial-temporal pyramid matching (STPM) kernel for comparison.

Figure 5a–c show classification accuracy of the proposed GPMK on Hollywood2, Youtube and HMDB51 datasets, respectively, and compare it with the baseline method (*i.e.*, STPM). We can see that our GPMK consistently outperforms the STPM kernel for each number of different maximum grid granularity levels (*i.e.*, *L* = 0, 1, 2, 3) on all of the three datasets. Concretely, the performance gain of the GPMK *w.r.t.* STPM is 1.4%–2.0% for the Youtube dataset and 5.0%–7.5% for the Hollywood2 dataset. For the more challenging HMDB51 dataset, our GPMK can obtain 3.6%–4.6% improvement of classification accuracy *w.r.t.* the STPM kernel. The superiority of GPMK demonstrates that it is able to compute better weights by utilizing the data-driven information from training samples and, thus, leads to a more discriminative video similarity metric for kernel-based SVM classification. Besides, as shown in Tables 1, 2 and 3, our method achieves superior classification accuracy compared to previous methods in action classification literature and reports the state-of-the-art results on these datasets.

### 5.4. Analysis and Discussion

**Figure 6.**
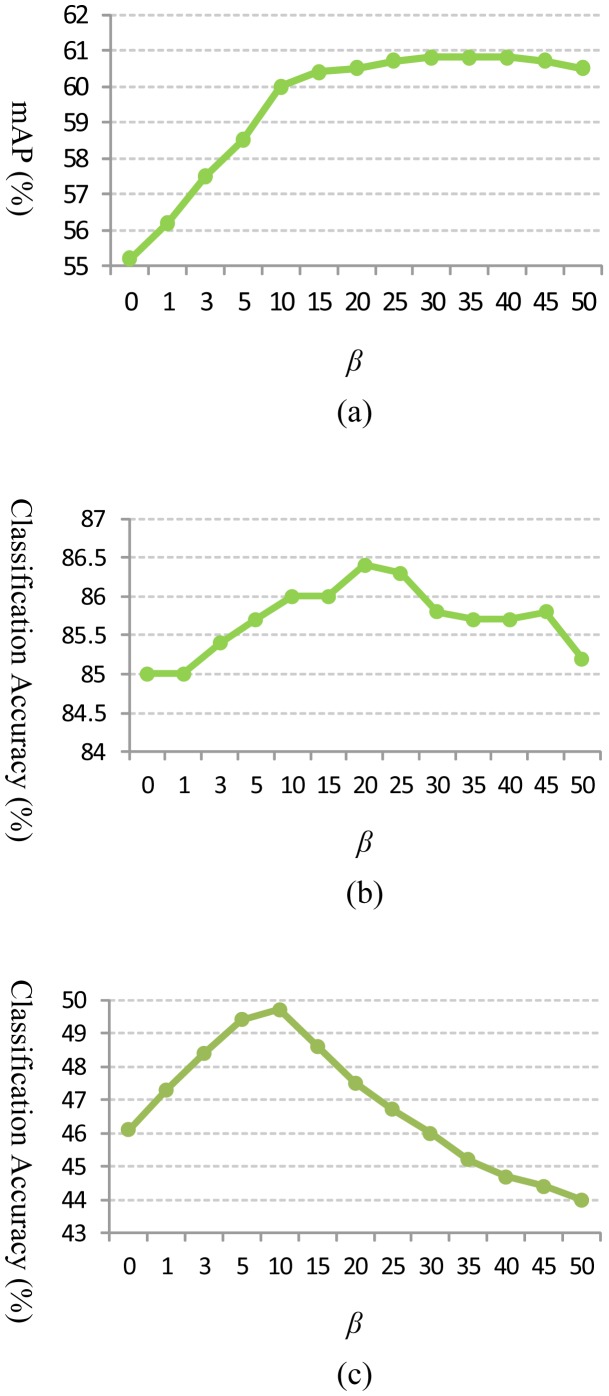
The recognition performance (shown on the Y-axis) *w.r.t.* different values of parameter *β* (shown on the X-axis) for GPMK: (**a**) Hollywood2; (**b**) Youtube; (**c**) HMDB51.

In this subsection, we provide detailed analysis and discussion on the proposed GPMK. As shown in Figure 5, we observe that the recognition performance achieves optimum with a 3-level pyramid (*i.e.*, *L* = 2) used for the GPMK, and thus, we set *L* = 2 for the discussion below. Figure 6 gives an empirical analysis on the effect of the smoothness parameter of soft max activation (i.e., *β*) for our GPMK. We can see that there exists an intermediate value of *β* for achieving optimum performance. When the value of *β* is too small, the weights of GPMK tend to be almost fully determined by the prior term and cannot make good use of the data-driven cues. Particularly, the GPMK will reduce to the baseline method if *β* = 0. On the contrary, the recognition performance of GPMK deteriorates, as well, when using too large a value for *β*. This is mainly because an excessively large value of *β* causes the weights to sharply concentrate on the channels with the largest kernel-target alignment values, and thus, the GPMK tends to utilize only a very small part of information from all the channels. This observation supports our motivation for combining prior knowledge and data-driven information, as in Equation (6).

**Figure 7.**
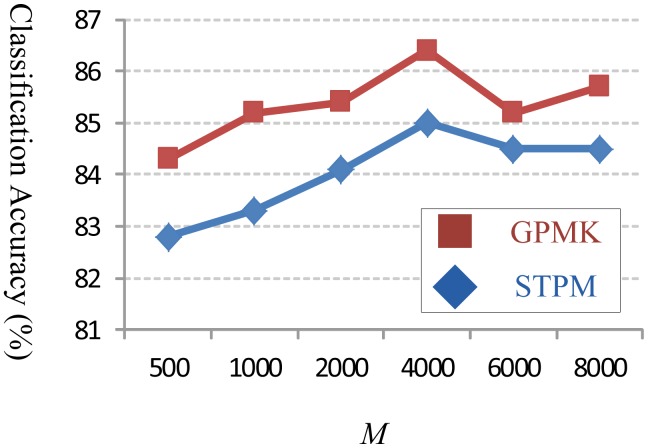
The classification accuracy (shown on the Y-axis) *w.r.t.* different values of *M* (shown on the X-axis) on Youtube dataset. The red square markers and the blue diamond ones correspond to our GPMK and the STPM kernel, respectively.

Moreover, we compare the GPMK with STPM for different numbers of visual words (*i.e.*, dictionary size *M*) used in experimental evaluation. As illustrated in Figure 7, our GPMK consistently outperforms the STPM kernel for each different value of *M*. This observation further validates the effectiveness of our method on computing adaptive channel weights in GPMK, and demonstrates its advantage *w.r.t.* the fixed and predefined weights used in STPM.

## 6. Conclusions

In this paper, we present a generalized pyramid matching kernel for human action recognition in realistic videos. It leverages heterogeneous visual cues in multiple feature descriptor types and spatial-temporal grid granularity levels, to build a valid video similarity metric for kernel-based SVM classification. Instead of the predefined and fixed weights used in SPM/STPM, we compute adaptive channel weights of GPMK according to the characteristic of training data. The experimental results on three public datasets validate the advantage of our GPMK *w.r.t.* the STPM kernel for human action recognition in realistic videos and show superior recognition performance compared to previous methods in literature.

## Figures and Tables

**Table 1.** Performance comparison of the proposed GPMK with previous methods in action classification literature (Hollywood2 [14]).

	**mAP** (%)
**Our Method**	**60.8**
Wang *et al.* [20]	59.9
Jiang *et al.* [34]	59.5
MIL-BoF [44]	48.73
L-MKL [46]	43.14
Le *et al.* [47]	53.3
Gilbert *et al.* [42]	50.9
Han *et al.* [18]	42.12
Marszalek *et al.* [14]	35.5

**Table 2.** Performance comparison of the proposed GPMK with previous methods in action classification literature (Youtube [15]).

	**Classification Accuracy** (%)
**Our Method**	**86.4**
Wang *et al.* [20]	85.4
MIL-BoF [44]	80.39
L-MKL [46]	77.91
Bhattacharya *et al.* [48]	76.5
Le *et al.* [47]	75.8
Human Postures [49]	77.8
Ikizler-Cinbis and Sclaroff [50]	75.21
Liu *et al.* [15]	71.2

**Table 3.** Performance comparison of the proposed GPMK with previous methods in action classification literature (HMDB51 [16]).

	**Classification Accuracy** (%)
**Our Method**	**49.7**
Wang *et al.* [20]	48.3
Jiang *et al.* [34]	40.7
MIP [51]	29.17
MIL-BoF [44]	31.53
Action Bank [19]	26.9
Kuehne *et al.* [16]	22.83
